# Application of imiquimod-induced murine psoriasis model in evaluating interleukin-17A antagonist

**DOI:** 10.1186/s12865-021-00401-3

**Published:** 2021-01-28

**Authors:** Qingran Li, Weiping Liu, Shidong Gao, Yao Mao, Yanfei Xin

**Affiliations:** Discovery Projects Unit, HitGen Inc, Building 6, No. 8 Huigu First East Road, Tianfu International Bio-Town, Shuangliu District, Chengdu, 610200 Sichuan China

**Keywords:** Psoriasis, Interleukin-17A, Imiquimod, Animal model, Autoimmune disease

## Abstract

**Background:**

Interleukin-17A (IL17A) is a proinflammatory cytokine critically involved in autoimmune diseases, and monoclonal antibodies of IL17A have been approved for clinical treatment of psoriasis. However, a usable psoriatic animal model has been always required for preclinical evaluation of IL17A antagonists. Imiquimod (IMQ)-induced psoriasis model is widely used in fundamental research, but it’s not able to accurately show anti-psoriatic effect of IL17A antagonists with conventional modelling condition.

**Results:**

On female C57BL/6 mice, with optimization on the usage of IMQ, positive control reagent and anti-mIL17A antibody, a 7-day model with proper testing window, acceptable disease severity as well as high repeatability was developed, and the efficacy of IL17A antagonist can be objectively evaluated by several qualitative and quantitative indices. Meanwhile, we validated the detailed involvement of IL17A signaling in disease progression, confirmed that the expression levels of IL17A and its related cytokines were induced by IMQ application, and its downstream cytokines can be inhibited by IL17A antagonist treatment. In further study, we revealed that IL17A was transient induced by IMQ and directly caused downstream signaling activation. This finding on the kinetical change of IL17A signaling will manifest the pharmacokinetics-pharmacodynamics investigation of IL17A antagonists.

**Conclusions:**

Our work presents the application of a convenient psoriatic animal model in the research and development of IL17A antagonists, meanwhile providing extra evidence for understanding IL17A’s role in the progression of IMQ-induced psoriasis model, which manifest the research and development of IL17A antagonists.

**Supplementary Information:**

The online version contains supplementary material available at 10.1186/s12865-021-00401-3.

## Background

Interleukin-17A (IL17A) is a member of IL17 family which mainly produced by T helper-17 cell and γδT cell, by acting as pro-inflammatory cytokine, it is critically participates in many inflammatory processes [1–3]. Involvement of IL17A in inflammation and autoimmune disease has been widely illustrated since its discovery on 2005, and abnormal expressions of IL17A are found in development of several diseases including psoriasis, rheumatoid arthritis and multiple sclerosis [4, 5]. By the findings of IL17A’s determinant role in diseases, feasibility of targeting IL17A for treating certain autoimmune diseases has been pursued since early 2010s. Up to date, Cosentyx (Novatis) and Taltz (Eli Lilly) are approved anti-IL17A monoclonal antibodies for clinical treatment of psoriasis, psoriatic arthritis and axial spondyloarthritis [6–8].

Psoriasis is a chronic autoimmune disease characterized by persistent, repeated happening of erythrosquamous plaques on skin, which overall affects 1% ~ 3% of people worldwide [9]. Involvement of IL17A in the pathogenesis of psoriasis has been thoroughly demonstrated [10], by directly activating IL17R of keratinocytes and triggering down-streamed signaling, IL17A induced expression of several pro-inflammatory cytokines and chemokines which recruit neutrophils and monocytes, and subsequently activate T-cells, finally causes hyperproliferation of keratinocyte and the generation of psoriasis [11]. Psoriasis is the first indication approved for clinical usage of anti-IL17A monoclonal antibody [12], and so far, psoriasis is still considered the very first indication for research of most IL17A antagonists.

For psoriasis research, there are several categories of experimental animal models, among which the “direct induction” type is most feasible for industrial drug development and screening [13, 14]. Imiquimod (IMQ) is a ligand for Toll-like receptors 7/8 which activates macrophages, monocytes and dendritic cells, by directly administrating on mice skin, it induces significant psoriasis-like skin damage [15, 16], and this model has been well-studied and widely used in fundamental research [17]. However, there are differences existing in both mechanism and clinical feature between IMQ-induced psoriasis in mice and human psoriasis [18], meanwhile in consideration of its acute and severe characters in disease progression, the usage of IMQ model is restricted in preclinical drug development.

Although using IMQ model in IL17A antagonist evaluation is not throughout investigated, the participation of IL17A in the model of IMQ-induced psoriasis has been reported by several studies. T cell produced IL17A is proved to be essential for development of disease, while blocking the IL17A signaling diminishes the severity of disease [19–23]. Although the exact involvement of IL17A in IMQ model remains to be comprehensively illustrated, its determinant role in disease development indicates this model is applicable for preclinical evaluation of IL17A antagonists.

On account of the requirement for a fast and stable psoriatic animal model to screen IL17A antagonists in our work, we optimized the conventional IMQ-induced psoriasis model, and developed a feasible modelling condition which gave a model with proper evaluation window and quantifiable/qualitative evaluation indices. Moreover, we provided extra information on the detailed involvement of IL17A signaling in disease development, which helps to illustrate PK-PD character of tested antagonists.

## Results

### Optimization of imiquimod-induced psoriasis model for illustrating the efficacy of IL17A antagonization

**Fig. 1:**
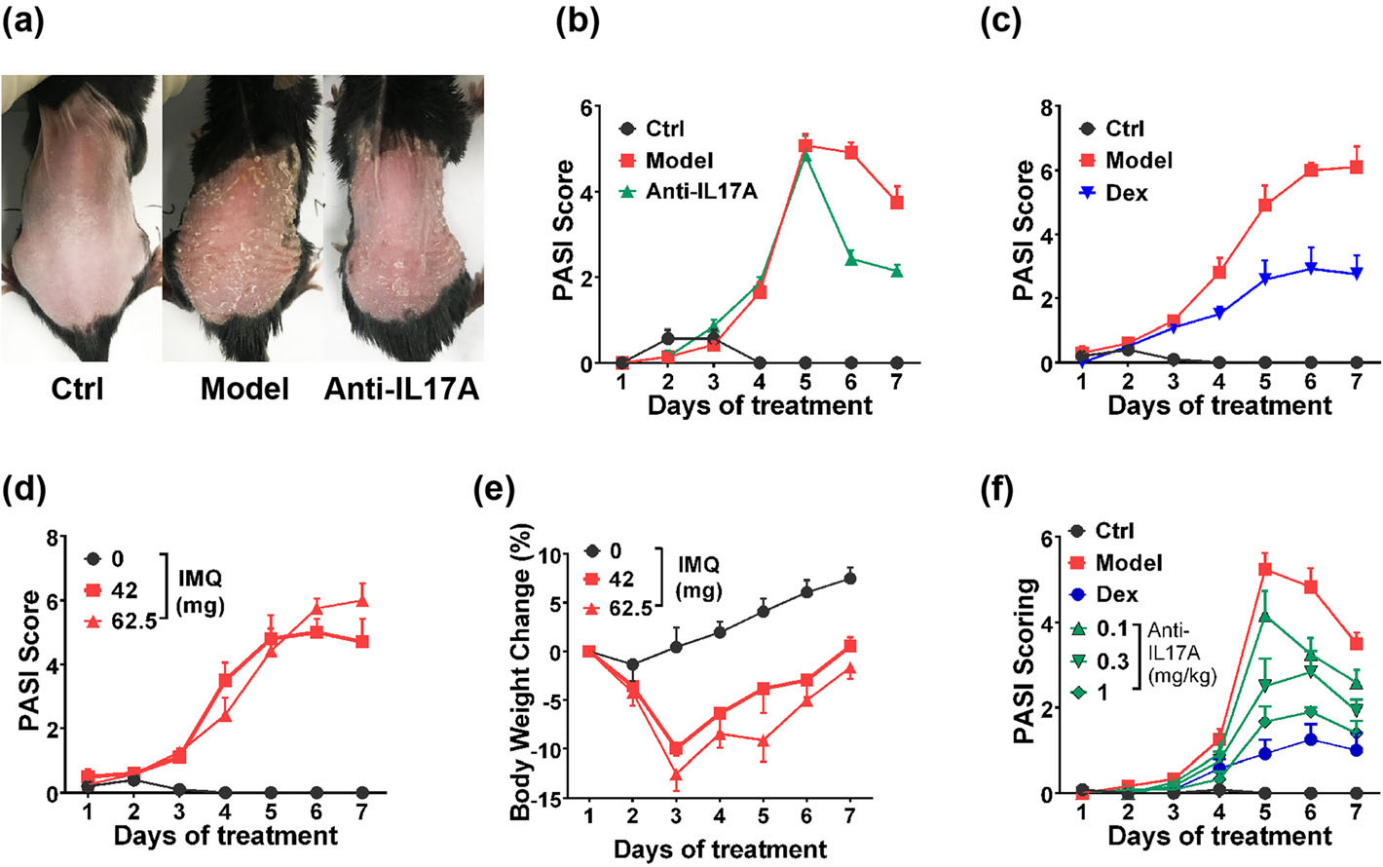
Optimization of Imiquimod (IMQ)-induced Psoriasis Model for Exhibiting Efficacy of Interleukin-17A Antibody on C57BL/6 Mice. **a** Skin damage (on day 5 of experiment) and **b** disease severity of 62.5 mg IMQ induced-psoriasis in mice. **c** Protective effect of Dexamethasone (10 mg/kg, daily) on 62.5 mg IMQ-induced psoriasis in mice. Effect of IMQ in different dosages on **d** disease severity and **e** bodyweight of mice. **f** Protective effect of Anti-IL17A antibody in different dosages (0.1–1 mg/kg) on IMQ (42 mg) induced psoriasis. “PASI Score” plot indicated disease severity evaluated by Psoriasis Area Severity Index (PASI) system (*n* = 6). Data was expressed as mean ± SEM, Anti-IL17A, Anti-IL17A antibody; Dex, Dexamethasone

Several researches have proved that consecutive 7-day application of 62.5 mg/mice/day IMQ cream on back skin induces significant psoriasis-like pathological change in C57BL/6 mice [18, 22, 24], we confirmed in our laboratory this modelling condition indeed induced classical pathological changes including the formation of white scales, thicken-induced skin wrinkle, as well as distinct redness on day 5 (Fig. 1a); However, blocking IL-17A pathway with anti-IL-17A antibody (10 mg/kg, 3 times/week) didn’t affect the disease process (only quicker drop in remission stage was observed, Fig. 1b). Therefore, the conventional modelling condition cannot illustrate the efficacy of IL17A antagonization, next we tried to optimize the model from several aspects.

IMQ-model is commonly developed in both C57BL/6 and BALB/c mice, but there is research indicating differences exist between these two strains [18], we tested whether antibody works on BALB/c mice. By using 62.5 mg/mice/day of IMQ as well, the disease was similarly induced with higher severity, and no distinct remission stage was observed (Fig. S1a). Nevertheless, anti-IL17 antibody still induced a minor protective effect in BALB/c mice; meanwhile, the sheet-shaped scaling lesion was easily fallen off during mice handling (Fig. S1b), which gained difficulty for PASI scoring. Therefore we still chose C57BL/6 over BALB/c mice in further optimization.

We introduced a positive reagent, dexamethasone (10 mg/kg, daily), for quality control of the model to exclude abnormal animal status or potential handling errors. Dexamethasone achieved a desirable therapeutic effect (Fig. 1c), which showed its suitability as a positive control, meanwhile proving no system error occurred during experiment.

There are reports indicating that IL17A pathway is not the only determinant in IMQ-induced mice psoriasis [19], we assumed that pathological change induced by 62.5 mg of IMQ was too severe to be protected by antibody. Interestingly, when evaluating the dose-dependency of IMQ (21, 42, 62.5 and 83 mg/day) on skin damage, the Psoriasis Area Severity Index (PASI) score showed that despite 21 mg of IMQ induced weak pathological changes, dosages of 42, 62.5 and 83 mg/day resulted in a similar extend of damage (Figs. 1d, S1c-d), and the change in skin thickness on day 7 of experiment indicated the same result (Fig. S1e). These finding indicated that 42 mg of IMQ is sufficient for inducing maximum severity, meanwhile suggesting that the dosage over 42 mg of IMQ induced IL-17A independent effect so that the antibody didn’t work well. By comparing the bodyweight change between different IMQ applications, dosages over 62.5 mg caused more serious weight loss and more death animals (Figs. 1e, s1f). Therefore, we chose 42 mg of IMQ as the suitable dosage for disease induction in current purpose.

To our expectation, antibody treatment showed a significant protective effect towards 42 mg IMQ modelling (Figs. 1f, S1g), we also noticed that the maximum efficacy of antibody was significantly weaker than dexamethasone. Since dexamethasone blocked the overall inflammation process, IL17A is likely to play a partial role in the pathogenesis of IMQ-induced psoriasis.

Beyond these factors, we tried to evaluate the minimal effective dosage of antibodies. IL-17A exposure in circulation after IMQ stimuli was limited to ~ 200 pg/ml level (Fig. 3b), for which 10 mg/kg antibody is far overdosed. In two individual experiments, we determined that dosages of antibody over 1 mg/kg induced comparable protective effects (Fig. S1g), while dosages of 0.1–1 mg/kg induced dose-dependent protection (Fig. 1f), so that 1 mg/kg of antibody was sufficient to achieve maximum effect. This clear dose-dependency of antibody strongly supported the participation of IL-17A signaling in this model.

In summary, with optimizations on animal strain, IMQ application and frequency/dosage of critical reagents, the model is now usable for evaluation of IL-17A antagonist.

### Characterization and evaluation of optimized model

**Fig. 2:**
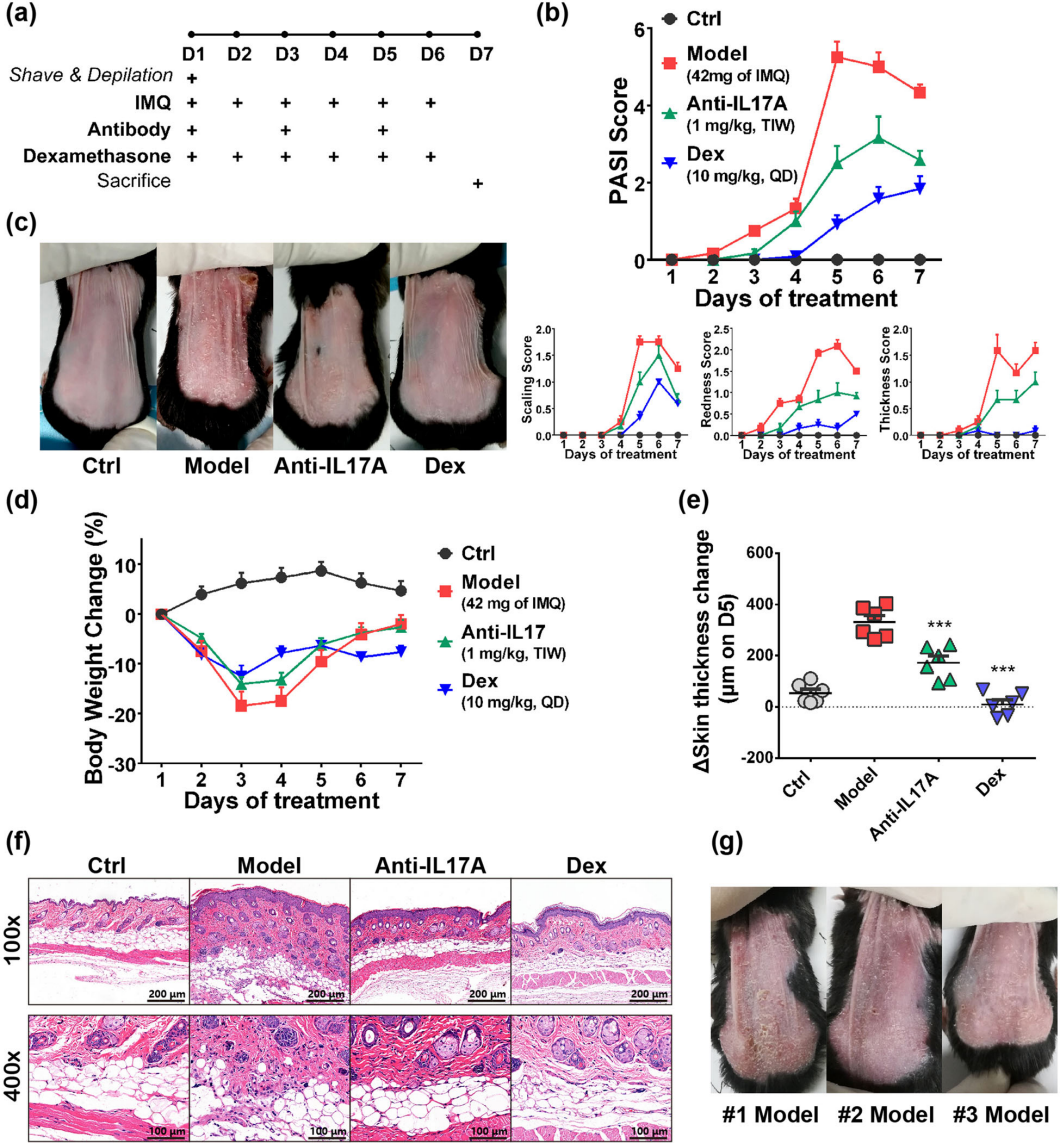
Characterization and Evaluation of Optimized IMQ-induced Psoriasis Model. **a** Graphic time-line of modelling. **b** Upper, typical PASI scoring plot for disease progression; lower, individual scoring for scaling formation, redness and thickness. **c** Representative images of back skin damage on day 5 of experiment. **d** Bodyweight change of mice from day 1–7. **e** Skin thickness change on day 5 of experiment. **f** Representative Hematoxylin-Erosin (H&E) staining of mice skin on day 7 of experiment. **g** Representative image of skin damage for model group from three different rounds of experiment. #1, #2 and #3 represents three individual experiment, respectively. Data was expressed as mean ± SEM (*n* = 6 or 7); *P < 0.05, ***P < 0.001 comparing to model group, Anti-IL17A, Anti-IL17A antibody; Dex, Dexamethasone

The timeline of experiment is illustrated in Fig. 2a. In the optimized condition, the model displayed a repeatable trend in pathogenesis (Fig. 2b). Briefly, the total PASI score of model group peaked on day 5 then gradually decreased on day 6–7, for each index of PASI scoring, redness gradually increased through modelling, while scaling and thickness robustly peaked on day 5 which was in accordance with the total score. The antibody group’s score had a similar tendency as model group, but to a lower level, while dexamethasone group didn’t have distinct score change throughout. The back skin appearance on day 5 (Fig. 2c) showed that uniform white scaling generated in mice from model group along with more redness and thicker skin wrinkles; the antibody had desirable, while dexamethasone had absolute protective effect on these pathological changes.

With the largest score window, PASI score on day 5 provides a proper quantitative index for evaluation of the protective effect as the severity of disease peaked (Fig. S2a); meanwhile, the Area under Curve (AUC) of PASI score can also be used to quantitatively compare disease severity and among groups (Fig. S2b). Notably, antibody treatment only caused 50% inhibition on PASI score and its AUC, suggesting that maximum efficacy of IL-17A antagonization was ~ 50% in this model.

The weight loss of mice also represents disease severity (Fig. 2d). Bodyweight of mice in model group has 20% decrease on day 3; this weight loss can partially be protected by the Anti-IL17A antibodies, and completely prevented by dexamethasone completely prevented the bodyweight decrease which is in accordance with the therapeutic effect. On day 5 of modelling, a generally a 2-fold increase in skin thickness occurred in model group (Fig. 2e); similarly to the PASI score and AUC, the reversion of thickness increase by anti-IL17A and dexamethasone were ~ 50% (*P* = 0.0009 vs model) and 100% (*P* = 0.0004 vs model), respectively.

IMQ induced typical psoriatic pathological changes in skin including thickening of the epidermal layer, parakeratosis, corium/fat layer, fat cell necrosis, fibrous hyperplasia as well as immune cell infiltration (Figs. 2f, S2c), these changes were partially reversed by the antibody, and vanished in dexamethasone group. Inflammatory cell significantly infiltrated in skin on IMQ treatment, which indicated the inflammatory signaling activation; the cell infiltration level was partially reversed by IL17A antibody and completely blocked by dexamethasone treatment (Fig. S2d).

Splenomegaly of mice is typical IMQ model which is frequently used to evaluate protective effect of test reagent. Interestingly, although dexamethasone perfectly reversed the gain of spleen weight, anti-IL17A treatment didn’t exert protective effect (Fig. S2e-f). We presumed IL17A antagonization cannot modulate IMQ-induced immune cell mobilization, therefore IL17A-dependent protective effect is not reflected by splenomegaly.

Repeatability of the model has been confirmed by several rounds of experiments. PASI score showed that development of disease was highly uniform among different experiments for each groups (Fig. S2g-h). The derivation of AUC and day 5 scoring (Table S1) and △skin (Table S2) thickness of each group was highly consistent among experiments which also proved the repeatability. Figure 2g shows the appearance of model group mice from 3 independent experiments on day 5, which also showed identical degree of psoriatic severity.

Together, these results illustrated the repeatability and reliability of this model, and also the suitability of this model for vertical comparison of different IL17 antagonists.

### Involvement of IL17A signaling in IMQ-induced psoriasis model

For better application of the model in preclinical small molecule IL17A inhibitor development, it’s necessary to understand the detailed involvement of IL17A in disease progression for designing of dosing strategy and mechanism of activity investigation. Therefore we probed into illustrating the exact role of IL17A and its signaling in IMQ-induced psoriasis progression.

**Fig. 3:**
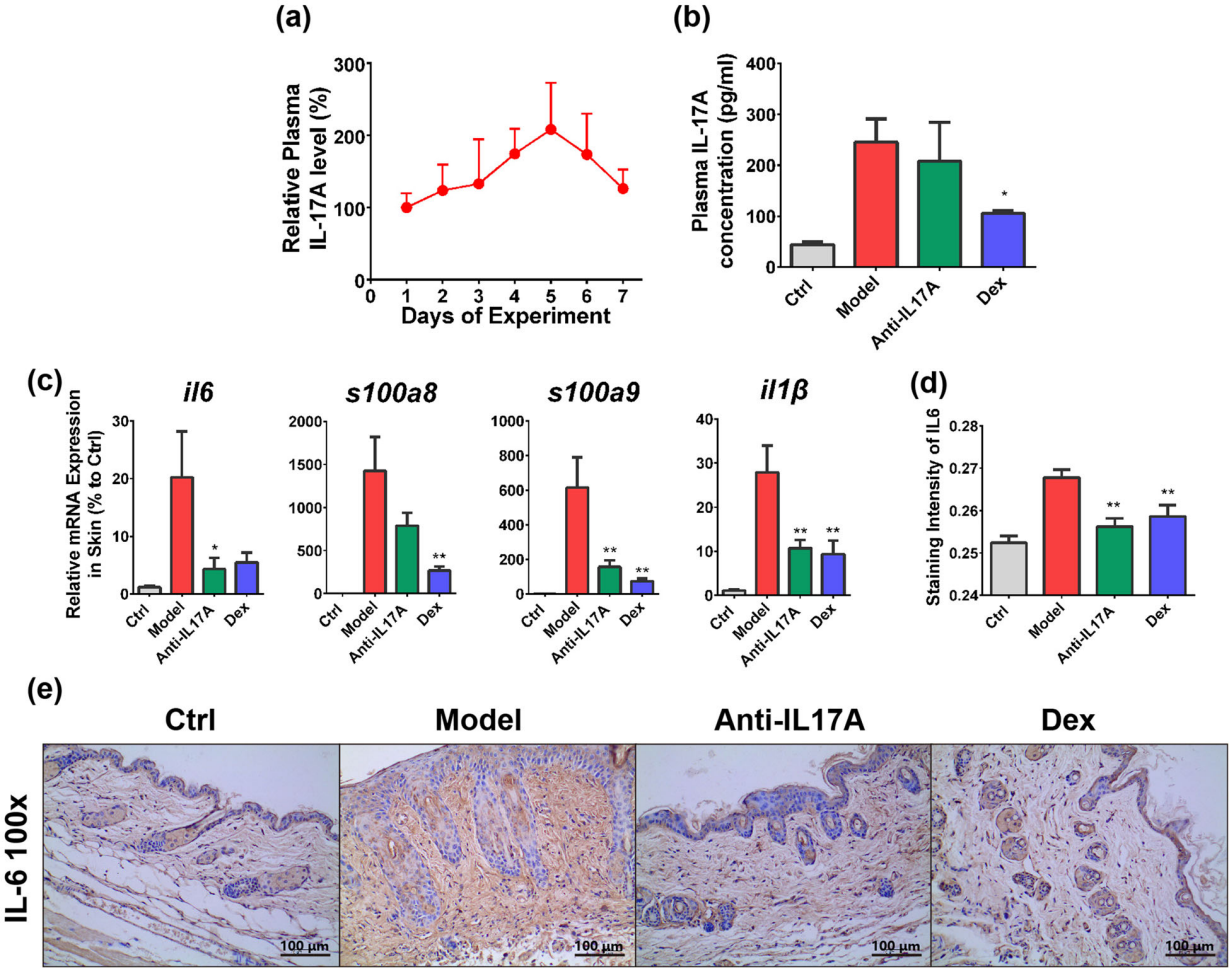
Involvement of IL17A Signaling in Disease Progression. **a** Plasma IL17A level during 7-day IMQ modelling (*n* = 4), from day 2 to day 7, plasma were collected 24 h after IMQ application on the previous day. **b** Plasma IL17A concentration of each group on day 5, 0.5 h after IMQ application (*n* = 6). **c** Expression level for mRNA of IL17A targeted genes in mice skin collected on day 5 after IMQ treatment (*n* = 6). **d** Staining intensity (*n* = 6) and **e** representative image of immunohistochemistry result for IL6 infiltration in mice skin on day 5. Data was expressed as mean ± SEM *P < 0.05, **P < 0.01 comparing to model group

We first determined the expression pattern of IL17A during disease development. After IMQ treatment, homeostatic level of IL17A gradually increased from the start of experiment, peaked on day 5 to about 2 fold, and decreased through day 6–7 (Fig. 3a), this trend was in accordance with pathological severity. On day 5, IL17A level was significantly suppressed by dexamethasone (*P* = 0.034) while not modulated by Anti-IL17A antibody (*P* = 0.873) treatment (Fig. 3b), suggesting antagonization of IL17A wouldn’t cause a feedback expression change of IL17A.

We selected day 5 for the downstream signaling study since maximum disease severity and IL17A level occurred on this day. Several inflammatory factors including IL6, IL1β, S100A8, S100A9 and CXCL1 are downstream effectors of IL17A, meanwhile playing an important role in the pathogenesis of IMQ model, by determining their mRNA expression pattern, we found that IMQ modelling significant upregulated mRNA level of these factors in skin, while antibody (*P* = 0.032, 0.002, 0.562, 0.008 for each gene) and dexamethasone (*P* = 0.062, 0.006, 0.008, 0.002 for each gene) distinctly reversed up regulation of *il6, il1β, s100a8 and s100a9* (Fig. 3c); however, as a classical cytokine for IMQ model, *cxcl1* was not regulated by both antibody and dexamethasone treatment, suggesting it’s insensitivity in current modelling condition (Fig. S3a). Similarly, we determined with immunohistochemistry that the infiltration of IL6 in the skin was also significantly inhibited by Anti-IL17A (*P* = 0.007) treatment (Fig. 3d-e), the reverse of IL6 level in skin by antibody further proved the efficacy of IL17A antagonization on preventing effector immune cell infiltration in skin. These results proved IL17A signaling was activated in the model, and indicated its involvement in disease progression.

### Regulation of IL17A signaling in the model and PD marker investigation

A sensitive PD marker is essential for confirming on-target effects, meanwhile helping to understand the potential PK-PD relationship of an IL17A antagonist. Plasma cytokines like IL6 are convenient PD markers for IMQ model, so we validated whether it’s usable for the current condition.

**Fig. 4:**
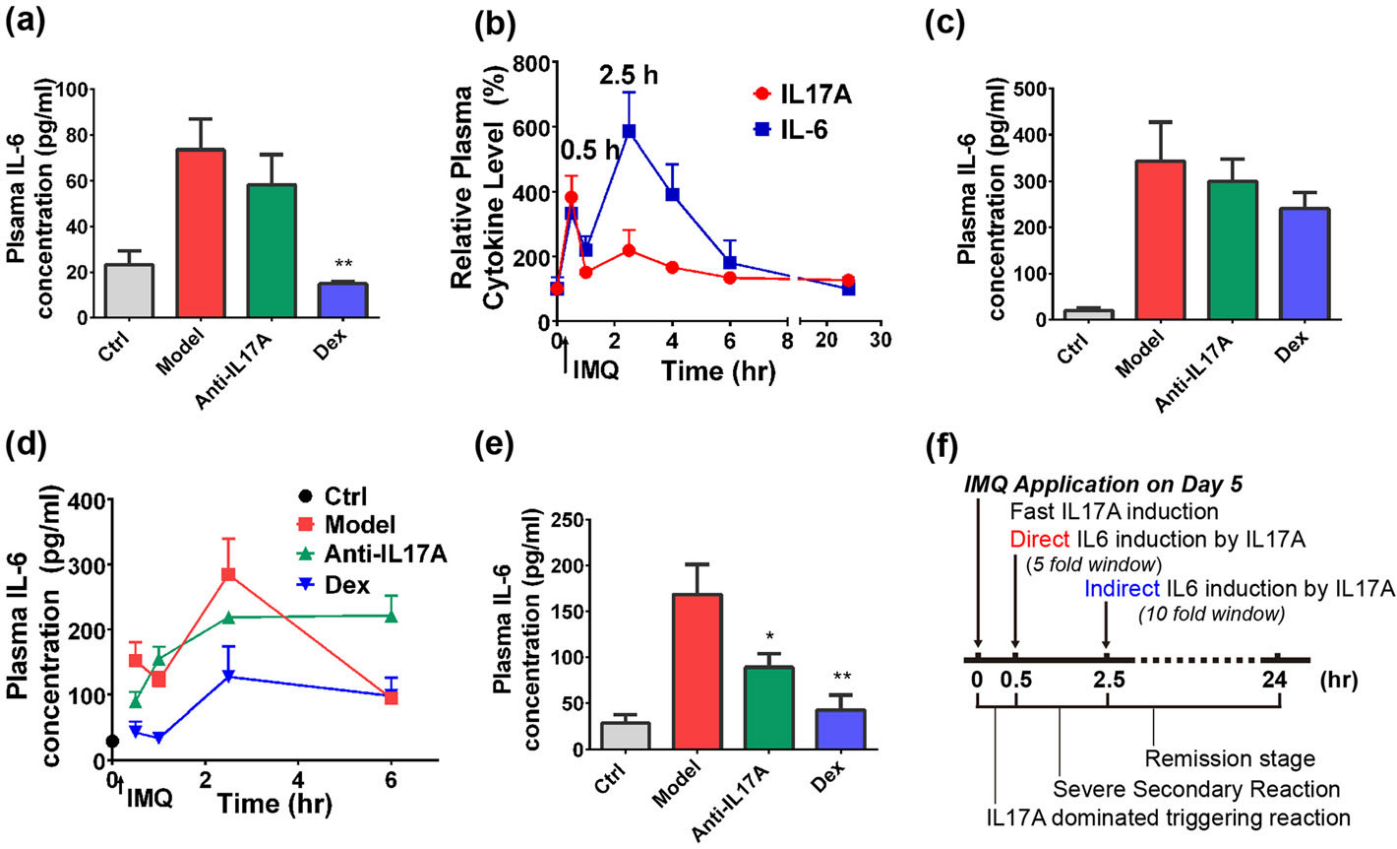
PD Marker Investigation and IL17A Kinetics in Disease Progression. **a** Plasma IL6 concentration on day 5 of experiment, 24 h after IMQ application of previous day (*n* = 6). **b** 24-h kinetical change of plasma IL17A and IL6 on day 5 of experiment after 42 mg IMQ application (*n* = 4). **c** Plasma IL6 concentration on day 5 of experiment, 2.5 h after IMQ application (*n* = 6). **d** Kinetical change of plasma IL6 for each group, 0–6 h after IMQ application on day 5 of experiment (*n* = 4). **e** Plasma IL6 concentration on day 5 of experiment, 0.5 h after IMQ application. **f** Time course of inflammatory signaling activation on day 5 of modelling. Data was expressed as mean ± SEM *P < 0.05, **P < 0.01 comparing to model group

On day 5 before IMQ application (24 h after IMQ treatment on day 4), despite the definite effect of dexamethasone (*P* = 0.003), Anti-IL17A didn’t significantly suppressed IL6 level in plasma (Fig. 4a); meanwhile, IMQ only induced low fold change of IL6 (2.5 fold) in model group, which was not consistent with previous reports [25]. We assumed IMQ might acutely induced IL6 level in plasma but decreased afterwards, so that the high fold-change cannot be observed after 24 h.

We performed a 24 h-monitor of IL6 on day 5. To our expectation, IL6 rapidly peaked 0.5 h after IMQ usage, then fastly decreased to the basal level on 1 h (Fig. 4b); interestingly, it peaked again on 2.5 h and reached a higher level, which was nearly 5-fold compared to the level before IMQ stimulation and ~ 10 fold to Control group (Fig. 4b), then decreased until 24 h; so we supposed the inhibitory effect of antibody on IL6 can be displayed this larger assay window on 2.5 h. Unexpectedly, at this time point antibody was still unable to show an inhibitory effect on IL6, and even dexamethasone didn’t exert significant inhibition (Fig. 4c).

We reconsidered the kinetics of IL6 and tried to find the answer from IL17A, wondering that besides the slow change in 7 days (Fig. 3a), whether IL17A has a kinetical change within 24 h. We noticed that after IMQ usage, IL17A peaked once on 0.5 h, which overlapped with the first peak of IL6 (Fig. 4b), this perfect synchronization suggested that IL6 elevation on 0.5 h was directly caused by IL17A, while the 2.5 h peak may be independent of IL17A signaling. To testify this assumption, we performed another kinetical monitoring of IL6 for both Control, model and treatment groups after IMQ usage. Within 0–6 h after IMQ treatment, IL6 of Anti-IL17A group was only significantly inhibited on 0.5 h, while gradually increased afterwards (Fig. 4d). Therefore, only the 0.5 h peak of IL6 was actually depended on IL17 and can be inhibited by Anti-IL17 treatment.

In summary, we concluded that IL17A was transiently induced 0.5 h after IMQ application, which directly triggered a synchronized IL6 elevation; while the second peak of IL6 was indirectly induced by IL17A thus didn’t response to antibody. We also compared the 24 h kinetic change of CXCL1 with IL17A (Fig. S3c), finding CXCL1 peaked on 2.5 h which overlapped with the second peak of IL6, explaining that CXCL1 is unable to act as an IL17A-related PD marker (Fig. S3a-b). By solely comparing IL6 levels of different groups on 0.5 h after IMQ application, the stimulated IL6 level was ~ 5 fold to control, this time Anti-IL17A treatment significantly inhibited IL6 induction (Fig. 4e, *P* = 0.039). Therefore plasma IL6 on 0.5 h after IMQ application was an applicable PD marker for IL17A antagonist evaluation.

## Discussion

In this study, we optimized the IMQ-induced mouse model of psoriasis and developed a repeatable model with proper severity and stable pathogenesis for the determination of IL17A antagonists. By consecutive 7-day-treatment with 42.5 mg of IMQ on female C57BL/6 mice, distinct psoriatic lesion was induced on the back skin with the severity peaked on day 5; disease progression and efficacy of the tested articles could be determined by several qualitative and quantitative indices. IL17A and its signaling in the model was further investigated on day 5, and the induction of IL17A downstream genes can be inhibited by Anti-IL17A treatment. By probing into the detailed expression pattern of IL17A in the model, we found besides the slow change through the 7 day modelling, IL17A was induced shortly after IMQ application to a high level, and directly caused synchronized IL6 elevation which can be used as PD marker for IL17A antagonist evaluation.

There are mainly four types of psoriasis models used in scientific research, including direct induction, spontaneous induction, genetically engineering and xenotransplantation. Except for direct induction, all models are characterized by high expense, long-term, high operative difficulty and low throughput which are not applicable for preclinical drug validation and screening [13, 14]. IMQ-induced psoriasis is a classical direct induction model and widely used in fundamental research and anti-psoriatic drug evaluation [20, 24, 26, 27]; there is also another direct induction model which intradermal inject IL23 in mice ear and induced local psoriasis like pathological change [28].

Participation of IL17A signaling in IMQ model has been revealed by previous reports, IMQ induced epidermal expression of IL17A during 6-day modelling, which is attributed to Th17 cell mobilization [22]; as a newly discovered receptor for IL17A, IL17RD is also determined for IMQ-induced psoriatic inflammation [23]; in another study, blocking IL17A signaling by knocking out IL17 Receptor A (IL17RA) diminishes the severity of IMQ induced pathological change [19]. IL17A expression is also positively correlated with the disease progression of the model: induction of IL17A by high-fat diet augments severity of disease, while impairing the IL17A-producing Th17 cells by vitamin D analogue exerts anti-psoriatic effect [20, 21]. Our study additionally revealed the involvement of IL17A in the development of IMQ-induced disease: despite of the slow change of basal level IL17A during the 7 day course of experiment, IMQ induced sharp IL17A expression after application, and the downstream signaling was correspondingly activated.

Nevertheless, Anti-IL17A treatment exerted inferior efficacy than glucocorticoids, indicating a “partial role” of IL17A signaling in IMQ induced psoriasis development. This “partial role” may explain the disability of using the conventional modelling condition to evaluate IL17A antagonists. Dosage of 62.5 mg IMQ has been extensively used in disease development of fundamental research, however, IL17A antibody didn’t show efficacy in this setting; By noticing that: 1) 42 mg and 62.5 mg of IMQ induced similar extent of skin damage, 2) bodyweight loss was more severe for 62.5 mg (Fig. 1e); and 3) Effect of 42 mg IMQ can be antagonized by antibody, we presumed IL17A independent effect induced by 62.5 mg of IMQ may conceal the real efficacy of antibody while cannot be reflected by PASI scoring. Therefore, IL17A related PD marker is essential to be evaluated when using this model to avoid the potential false positive result.

By illustrating plasma IL6 kinetics and its relationship with IL17A, we determined the availability of IL6 as PD marker to illustrate the IL17A signaling status [29–31], by which the successful antagonization of IL17A resulted in distinct IL6 suppression 0.5 h after IMQ application. The kinetical character of IL17A/IL6 activation in this model is summarized in Fig. 4f. On day 5 of experiment, IL17A was transient activated on 0.5 h after IMQ application, while IL6 was twice induced on 0.5 and 2.5 h; the 0.5 h peak of IL6 was dependent on IL17A since it synchronized with the IL17A activation and can be suppressed by IL17A antagonizing. We presumed the 0.5 h activation of IL17A/IL6 acted as triggering signal, and the bottom of IL6 level on 1 h showed a clear “preparing phase” of immune cell mobilization and signaling transduction, the eventually 2.5 h induction IL6 directly caused psoriatic damage. In further study, consistency of this effect on different day of IMQ application is interesting to be illustrated.

Revealing and understanding the kinetics of IL17A was critical for application of the model in IL17A antagonist development. Each usage of IMQ is likely to induce IL17A elevation, since antibody usually possesses longer half-life in vivo [26], the 3-times-per-week frequency is sufficient to antagonize daily induced IMQ effect. However, small molecule antagonists generally eliminate faster in vivo, based on their PK-PD character, it’s important to design rationally pre-dosing strategy via a suitable route to achieve plasma exposure, so that level of IL17A at the robust stage is fully covered, and efficacy of antagonist can be correctly exhibited. Our efforts also provide an example for mechanism-directed model optimization, illustrating that instead of using a generally applicable model, balancing modelling condition based on the research purpose and pathogenesis mechanism achieves a suitable model which promotes the data quality and efficiency of research.

Other highlights of the optimized model include less adverse effects and higher repeatability. Drastic inflammatory effect is induced by IMQ, mice then experience severe dehydration and associated bodyweight loss, which cause weakness even death during the experiment. When using 62.5 mg IMQ, generally 6 ~ 8% mice dead on day 3-day 5 was observed; while 42 mg IMQ was better tolerated by mice and rare death of mice happened. Psoriatic damage is also induced by IMQ in the ear of mice in many studies [22, 23, 32, 33], we didn’t considered the ear index in our practice so that extra usage of IMQ and related adverse effect can be avoided. These attempts on reducing the adverse effects also contributed to the reproducibility of model (Figs. 2g, S2g-h, Table S1–2), the degree of pathological damage was also identical among each round of experiment. A stable and repeatable model is undoubtedly essential for antagonists’ evaluation, as well as the comparison of the same series of test articles in different rounds of experiment.

This study deepened the research of IMQ-induced psoriasis model, fully illustrating the dose-dependent phenotype and strategy of avoiding adverse effect; meanwhile, it broadens the understanding of IL17A in psoriasis pathogenesis, as well as modulating IL17A pathway for treatment of psoriasis. Finally, with the convenient modelling condition and clear dependency on IL17A signaling, this model provides effective tool for preclinical screening of IL17A antagonists, strongly supporting area IL17A-related drug development.

## Conclusions

Our study optimized classical IMQ-induced psoriasis mouse model and provided a highly repeatable model with abundant qualitative/quantitative evaluation indices. By showing the involvement of IL17A signaling in the model and illustrating its capability in performing PK-PD investigation of IL17A antagonists, this model is suitable for fast screening and evaluating of IL17A antagonist in preclinical stage. We hope this model manifests scientists and pharmaceutical industries for development of IL17A antagonists in the future.

## Methods

### IMQ-induced psoriasis-like model

All animal related protocols were reviewed and approved by Animal Welfare Ethics Committee of Hitgen Inc., and all methods were carried out in accordance with relevant guidelines and regulations.

Animal related experiments are conducted in animal facility in Hitgen Inc. Female C57BL/6 mice or BALB/c mice aged 10 weeks were purchased from Vital River (Beijing, China). Mice were adaptively cultured for at least 3 days on arrival and kept in specific-pathogen free environment with free access to water and food. For IMQ-induced psoriasis, mice were shaved and completely unhaired with depilation cream on the back with 2*3 cm of size on day 1; indicated amount of Vaseline (Panyan Biotech, Jiangsu, China) or 5% IMQ cream (Med-Shine Pharmaceutical, Sichuan, China) were applied on exposed back skin for 7 consecutive days (for severity monitoring), or 5 days (for PD study); positive control reagents (Dexamethasone Sodium Phosphate Injection, CSPC, Hebei, China; InVivoMab Anti-Mouse IL17A, BioXcell, NH, USA) were intraperitoneally administrated 1 h before IMQ application on each dosing day, and weight of mice was daily recorded.

Samples and several critical parameters indices were obtained on day 5 of experiment. Mice were scored, photographed on the back skin, and then dosed with the test articles; IMQ creams were applied on the back skin after indicated time interval to induce instant inflammatory reaction; plasma of mice was collected 0.5 h after IMQ application for cytokine determination, mice were hereupon sacrificed and ~ 1 cm^2^ of back skin were fixed in buffered 4% paraformaldehyde solution for histopathological study; and ~ 50 mg of skin were instantly frozen in liquid nitrogen for followed RT-PCR experiment.

### PASI scoring system and bodyweight

The severity of psoriasis-like change was daily evaluated according to modified PASI scoring system [34]. Factors of erythema, scaling and thickening of back skin were quantified on range from 0 to 4 point (0, no change; 1, mild change; 2, marked change; 3, significant change; 4, severe change), the PASI score in figure represented the sum of each factors in the scoring day. Referring to Figure S4 for representative images of psoriatic back skin with different severities and the detailed scoring information.

The bodyweight of the animal was recorded daily, any animal with sudden loss of bodyweight > 20% was excluded from IMQ application on that day. Bodyweight change was calculated by weight change = (Weight_dn_ – Weight_day 1_)/Weight_day 1_ *100%.

### Determination of back skin thickness

Back skin thickness was measured by Vernier caliper on day 1, day 5 or day 7 of experiment. Mice were held by one operator to expose the back skin, and another operator randomly picked 3 positions on the skin to perform measurement, double-layer thickness was recorded. For each mouse, skin thickness change = average (Th_dn_) – average (Th_day 1_).

### Inflammatory factors determination

Whole blood of mice were collected in EDTA-K2 precoated centrifuge tube, and plasma were separated and stored at − 20 °C before determination. Cytokines of IL6 and IL17A in plasma were determined via the classical ELISA method according to the instruction of manufacturer. Duoset ELISA kits were purchased from Biolegend (CA, USA), plate were coated overnight with antibody before usage.

### Histopathological and immunohistochemical study

Histopathological and IL6 immunohistochemical determination were performed by Lilai Biotechnology (Sichuan, China). Briefly, the fixed skin samples were dehydrated, embedded, sectioned and stained. The result pathological samples were analyzed and photographed by DM-BA200 microscope (Motic, BC, Canada), 100× and 400× images of typical position were taken for each sample. Hematoxylin and eosin (H&E) staining was applied to determine histopathological change, the scoring were mainly based on pathological changes including thickening of the epidermal layer, parakeratosis, corium/fat layer immune cell infiltration, fat cell necrosis and fibrous hyperplasia, each item was scored based on microscopic check and graded from 0 to 4 points (0, normal; 1, mild; 2, low; 3, medium; 4, severe), total H&E pathological represented the summation of each item. IL6 expression was determined by optical density of DAB labeled IL6 antibody (Proteintech, IL, US) staining.

### RNA reverse transcription and real-time PCR

Skin sample of 50 mg for mRNA expression analysis was pre-minced before instant freeze, collected in centrifuge tube, and stored at − 80 °C before operation. For total RNA extraction, 1 ml of Trizol Reagent (Beyotime, Jiangsu, China) were added in to the sample, the sample was mechanically grinded, and extracted by 0.2 ml of chloroform; after centrifuge, the upper layer solution was transferred and total RNA was precipitated by isopropanol and purified by 75% ethanol. RNA reverse transcription was performed using EasyScript One-Step gDNA Removal and cDNA Synthesis Supermix kit (TransGen, Beijing, China) according to the instructions of manufacturer for acquiring cDNA. Then Real-Time PCR was performed using PowerUp™ SYBR™ Green Master Mix (Applied Biosystems, VA, USA) in 384 plate with Quantstudio™ 6 Flex System (Applied Biosystems). Primers were synthesized by Sangon Biotech (Shanghai, China). Information of primers for analyzed genes are illustrated in Table S3. Data was analyzed by Livak method to determine difference of gene expression from samples. Briefly, C_T_ value of target gene was normalized by C_T_ value of β-actin to acquire C_T(Norm)_, C_T(Norm)_ of model/treatment groups were calibrated by C_T(Norm)_ of Control group; Finally, 2^-Cal[CT(Norm)]^ was calculated to determine the relative gene expression.

### Statistical analysis

Data was presented as mean ± S.E.M. For multiple groups comparison of determined values (skin thickness, spleen weight, gene expression, cytokine level), one-way ANOVA followed by LSD or Dunnett’s T3 was used after determination of homogeneity of variance; for scoring values (PASI Scoring, AUC of PASI scoring and Histopathological scoring), non-parametric testing (Kruskal-Wallis) of independent samples were used, *P* < 0.05 was considered significantly different.

## Supplementary Information

**Additional file 1: Figure S1.** Optimization of Imiquimod (IMQ)-induced Psoriasis Model for Exhibiting Efficacy of Antibody. **Figure S2** Evaluation of Optimized Psoriasis Model. **Figure S3.** Involvement of CXCL1 in IMQ-induced Psoriasis Progression. **Figure S4** Representative Images for PASI Scoring. **Table S1.** Variation of Psoriasis Area Severity Index (PASI) score on day 5 and area under curve (AUC) of PASI score for Model, Anti-IL17A and Dexamethasone (Dex) groups among three independent rounds of experiment. **Table S2.** Variation of skin thickness change for model group among three independent rounds of experiment. **Table S3.** Information of RT-PCR Primer for Analyzed Gene.

## Data Availability

The datasets generated and/or analyzed during the current study are not publicly available due to privacy restrictions but are available from the corresponding author on reasonable request.

## Abbreviations

Th17 cells: T-helper 17 cells
TLR: Toll-like Receptor
IL17A: Interleukin-17A
IL17R: Interleukin-17 Receptor
IL6: Interleukin-6
S100A8/A9: S100 Calcium Binding Protein A8/A9
CXCL1: Chemokine (C-X-C motif) ligand 1
IMQ: Imiquimod
PK: Pharmacokinetics
PD: Pharmacodynamics
PASI: Psoriasis Area Severity Index
AUC: Area Under Curve
