# Supplementary material for: Application of imiquimod-induced murine psoriasis model in evaluating interleukin-17A antagonist
Source: BMC Immunol. 2021 Jan 28;22:11. doi: 10.1186/s12865-021-00401-3 (PMC7844923; doi:10.1186/s12865-021-00401-3)
Supplement: Supplementary file 1 — Additional file 1: Figure S1. Optimization of Imiquimod (IMQ)-induced Psoriasis Model for Exhibiting Efficacy of Antibody. Figure S2 Evaluation of Optimized Psoriasis Model. Figure S3. Involvement of CXCL1 in IMQ-induced Psoriasis Progression. Figure S4 Representative Images for PASI Scoring. Table S1. Variation of Psoriasis Area Severity Index (PASI) score on day 5 and area under curve (AUC) of PASI score for Model, Anti-IL17A and Dexamethasone (Dex) groups among three independent rounds of experiment. Table S2. Variation of skin thickness change for model group among three independent rounds of experiment. Table S3. Information of RT-PCR Primer for Analyzed Gene. [file 12865_2021_401_MOESM1_ESM.docx]

Supplementary Information for

**Application of Imiquimod-induced Murine Psoriasis Model in Evaluating Interleukin-17A Antagonist**

Qingran Li^1^, Weiping Liu^1^, Shidong Gao^1^, Yao Mao^1^, Yanfei Xin^1,*^

^1^Discovery Projects Unit, HitGen Inc, Building 6, No. 8 Huigu First East Road, Tianfu International Bio-Town, Shuangliu District, Chengdu 610200, Sichuan, China.

*Correspondence author: Dr. Yanfei Xin, E-mail: [yf.xin@hitgen.com](mailto:yf.xin@hitgen.com)

**Supplemental Figures**

**
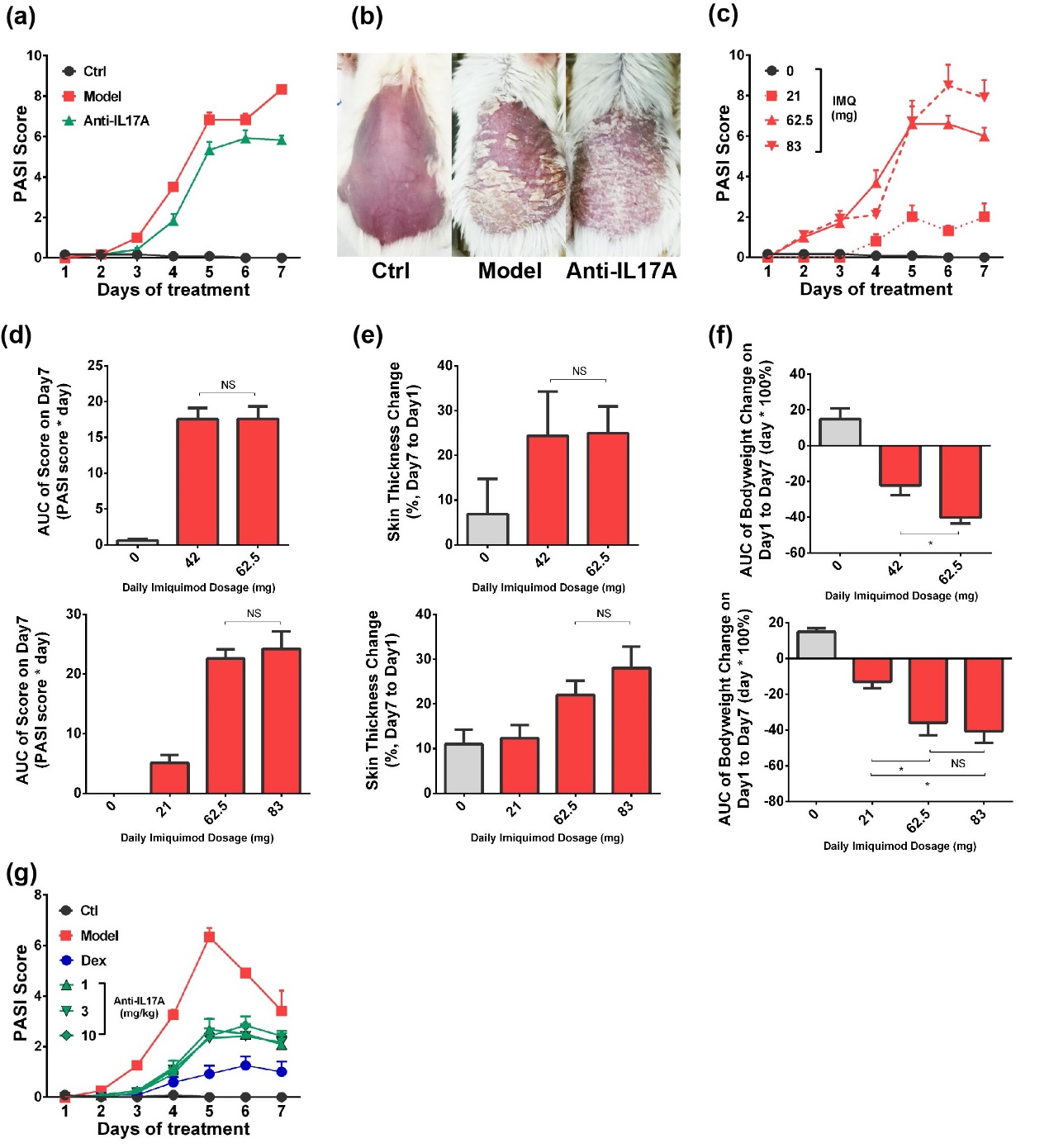
**

Figure S1 **Optimization of Imiquimod (IMQ)-induced Psoriasis Model for Exhibiting Efficacy of Antibody.** (a) Disease severity and (b) skin damage (day 5) of 62.5 mg IMQ induced-psoriasis in BALB/c mice. Effect of IMQ in different dosages on (c) disease severity, (d) AUC of PASI scoring, (e) skin thickness change and (f) AUC of bodyweight change (day 1-day 7). (g) Protective effect of Anti-IL17A antibody in different dosages (1-3 mg/kg) on IMQ (42 mg) induced psoriasis, Data was expressed as mean ± SEM.


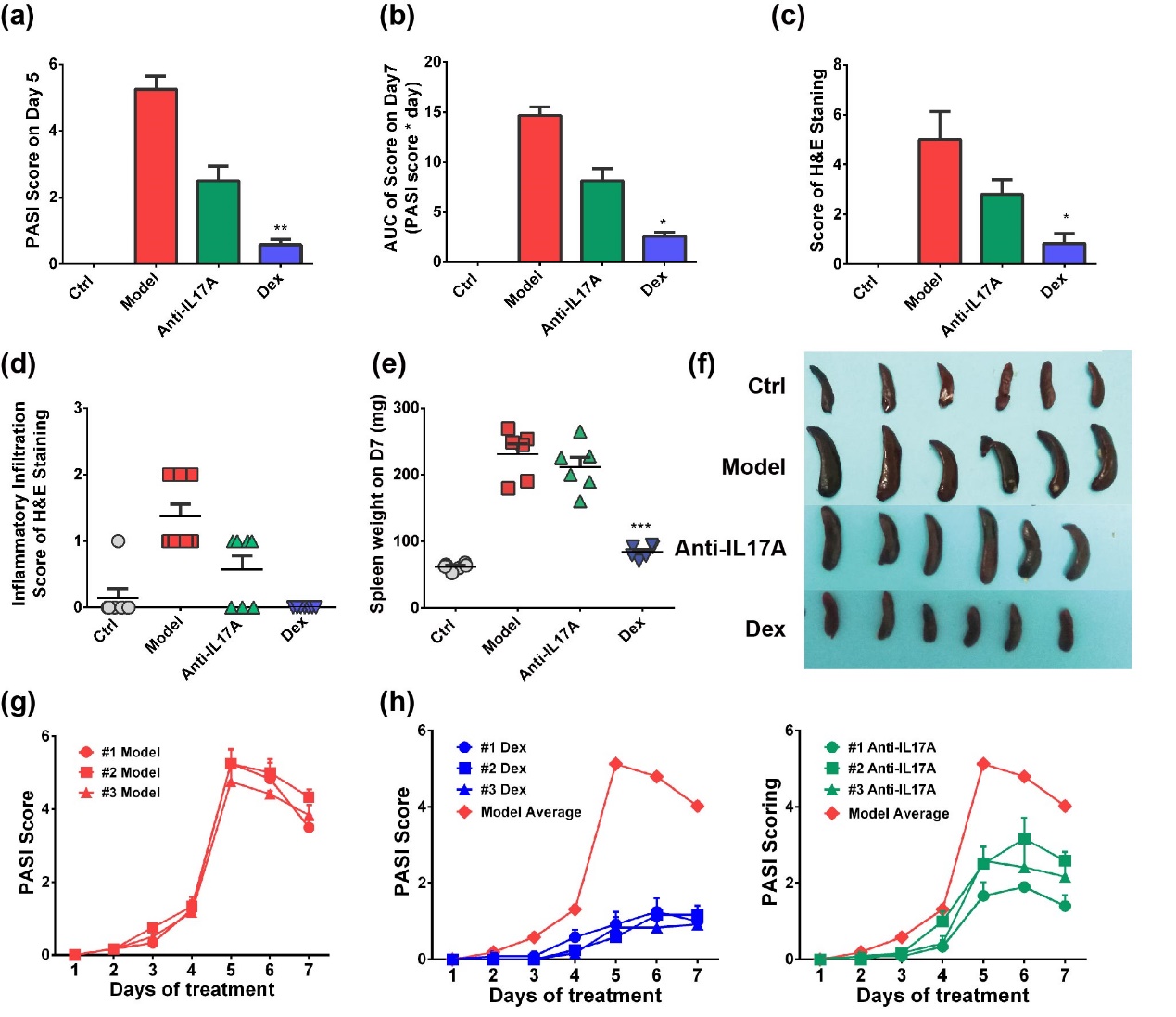


Figure S2 **Evaluation of Optimized Psoriasis Model.** Comparison of (a) PASI score on day 5 and (b) Area under curve (AUC) for PASI score plot for day 1-7 of experiment (n=6). (c) Total H&E scoring and (d) H&E-based inflammatory infiltration scoring of mice on day 7 of experiment. (e) Spleen weight and (f) size on day 7 of experiment (n=6). PASI score for (g) Model group and (h) Dexamethasone/Anti-IL17A antibody group from different rounds of experiment. #1, #2 and #3 represents three individual experiments, respectively (n=6 or 7). Data was expressed as mean ± SEM.


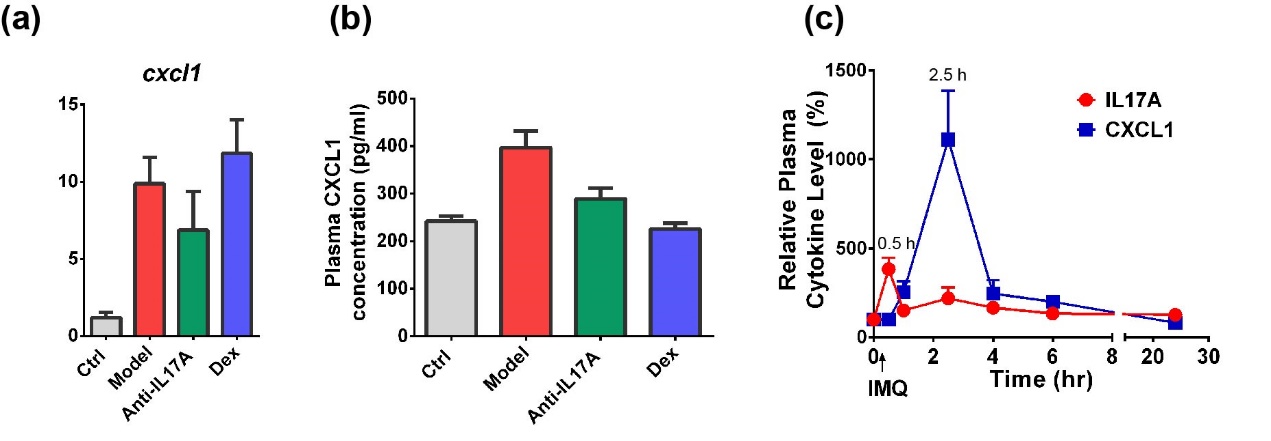


Figure S3 **Involvement of CXCL1 in IMQ-induced Psoriasis Progression.** (a) Expression level of *cxcl1* mRNA in mice skin collected on day 5 after IMQ treatment (n=6). (b) Plasma IL6 concentration on day 5 of experiment, 0.5 h after IMQ application (n=6). (c) 24-h kinetical change of plasma IL17A and CXCL1 on D5 after 42 mg IMQ application (n=4).


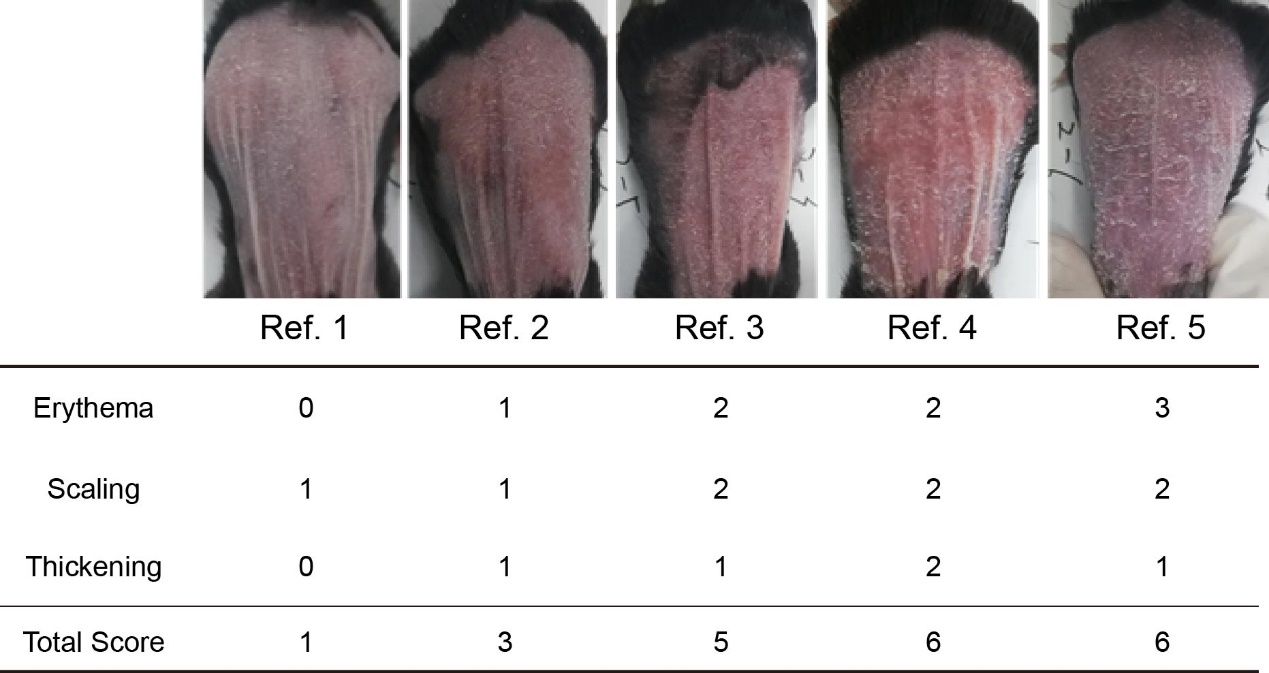


Figure S4 **Representative Images for PASI Scoring.** Ref. 1-Ref. 5 are representative back skin images from a single round of experiment on day 5, of which the detailed scoring information for erythema, scaling and thickening are listed.

**Supplemental Tables**

Table S1 Variation of Psoriasis Area Severity Index (PASI) score on day 5 and area under curve (AUC) of PASI score for Model, Anti-IL17A and Dexamethasone (Dex) groups among three independent rounds of experiment. Data were represented as means ± SEM, RSD: Relative Standard Deviation.

| **Group** | **Score on Day 5** | | | **RSD (%)** | **Total AUC** | | | **RSD (%)** |
| --- | --- | --- | --- | --- | --- | --- | --- | --- |
| Model | 5.08 | ± | 0.17 | 5.68 | 13.72 | ± | 0.51 | 6.44 |
| Anti-IL17A | 2.25 | ± | 0.29 | 22.53 | 6.40 | ± | 1.11 | 24.30 |
| Dex | 0.78 | ± | 0.10 | 22.30 | 2.76 | ± | 0.34 | 21.12 |

Table S2 Variation of skin thickness change on day 5 for model group among three independent rounds of experiment. Data were represented as means ± SEM, RSD: Relative Standard Deviation.

| **Group** | **ΔSkin Thickness (μm) of Model on Day 5** | | | **RSD (%)** |
| --- | --- | --- | --- | --- |
| Model | 336.67 | ± | 23.00 | 5.68 |

Table S3 Information of RT-PCR Primer for Analyzed Gene

| **Gene** | **Forward (5’-3’)** | **T_m_ (℃)** |  | **Reversed (5’-3’)** | **T_m_ (℃)** |
| --- | --- | --- | --- | --- | --- |
| *β-actin* | CCATGTACCCAGGCATTGCT | 60.40 |  | GTGTAAAACGCAGCTCAGTAACA | 59.75 |
| *il6* | GTCCTTCCTACCCCAATTTCCA | 59.69 |  | TAACGCACTAGGTTTGCCGA | 59.68 |
| *il1β* | GACTCCTTAGTCCTCGGCCA | 60.68 |  | GCTGCCTTAGTCCTCGGCCA | 59.74 |
| *s100a8* | ACAAGGAAATCACCATGCCC | 58.44 |  | TGAGATGCCACACCCACTTT | 59.52 |
| *s100a9* | GCTGCATGAGAACAACCCAC | 59.76 |  | TCCCTTTAGACTTGGTTGGGC | 59.93 |
| *cxcl1* | TGGCTGGGATTCACCTCAAG | 59.67 |  | CCGTTACTTGGGGACACCTT | 59.60 |
